# Loss of SAV1 in Kidney Proximal Tubule Induces Maladaptive Repair after Ischemia and Reperfusion Injury

**DOI:** 10.3390/ijms25094610

**Published:** 2024-04-23

**Authors:** Daeun Moon, Babu J. Padanilam, Kwon Moo Park, Jinu Kim

**Affiliations:** 1 Department of Anatomy, Jeju National University College of Medicine, Jeju 63243, Republic of Korea; qjxms9312@jejunu.ac.kr; 2 Department of Urology, Tisch Cancer Institute, Icahn School of Medicine at Mount Sinai, New York, NY 10029, USA; babu.padanilam@mountsinai.org; 3 Department of Anatomy, BK21 Plus, and Cardiovascular Research Institute, School of Medicine, Kyungpook National University, Daegu 41944, Republic of Korea; kmpark@knu.ac.kr; 4 Interdisciplinary Graduate Program in Advanced Convergence Technology & Science, Jeju National University, Jeju 63243, Republic of Korea

**Keywords:** Hippo signaling pathway, salvador family WW domain containing protein 1, ischemia and reperfusion injury, kidney regeneration, protein kinase B

## Abstract

Kidney ischemia and reperfusion injury (IRI) is a significant contributor to acute kidney injury (AKI), characterized by tubular injury and kidney dysfunction. Salvador family WW domain containing protein 1 (SAV1) is a key component of the Hippo pathway and plays a crucial role in the regulation of organ size and tissue regeneration. However, whether SAV1 plays a role in kidney IRI is not investigated. In this study, we investigated the role of SAV1 in kidney injury and regeneration following IRI. A proximal tubule-specific knockout of *SAV1* in kidneys (*SAV1*^ptKO^) was generated, and wild-type and *SAV1*^ptKO^ mice underwent kidney IRI or sham operation. Plasma creatinine and blood urea nitrogen were measured to assess kidney function. Histological studies, including periodic acid-Schiff staining and immunohistochemistry, were conducted to assess tubular injury, SAV1 expression, and cell proliferation. Western blot analysis was employed to assess the Hippo pathway-related and proliferation-related proteins. SAV1 exhibited faint expression in the proximal tubules and was predominantly expressed in the connecting tubule to the collecting duct. At 48 h after IRI, *SAV1*^ptKO^ mice continued to exhibit severe kidney dysfunction, compared to attenuated kidney dysfunction in wild-type mice. Consistent with the functional data, severe tubular damage induced by kidney IRI in the cortex was significantly decreased in wild-type mice at 48 h after IRI but not in *SAV1*^ptKO^ mice. Furthermore, 48 h after IRI, the number of Ki67-positive cells in the cortex was significantly higher in wild-type mice than *SAV1*^ptKO^ mice. After IRI, activation and expression of Hippo pathway-related proteins were enhanced, with no significant differences observed between wild-type and *SAV1*^ptKO^ mice. Notably, at 48 h after IRI, protein kinase B activation (AKT) was significantly enhanced in *SAV1*^ptKO^ mice compared to wild-type mice. This study demonstrates that *SAV1* deficiency in the kidney proximal tubule worsens the injury and delays kidney regeneration after IRI, potentially through the overactivation of AKT.

## 1. Introduction

Kidney ischemia and reperfusion injury (IRI) is a significant contributor to acute kidney injury (AKI), leading to elevated mortality and morbidity rates [1,2]. Histopathologically, AKI is characterized by tubular injury, which include the loss of brush border, mainly in the proximal tubular epithelium, along with epithelial cell sloughing and cell death in the kidneys [3,4]. Unlike other organs, IRI-damaged kidneys have an excellent recovery ability; however, if the damage is severe, their recovery capacity rapidly diminishes [4,5]. The mechanisms involved in the kidney repair process include repopulation of the damaged tubules through cell regeneration [4]. After kidney IRI, surviving tubular cells have the ability to regenerate and repair the damaged kidney tubules [6,7]. Complete kidney function repair involves the restoration of kidney tubules, the improvement of glomerular filtration rate, and the recovery of overall kidney function [8,9]. However, incomplete recovery from AKI, also known as maladaptive repair, can result in long-term functional deficits and contribute to the development of chronic kidney disease (CKD) [10]. The transition from adaptive to maladaptive kidney repair in AKI that leads to CKD can be attributed to tubular atrophy and degeneration, the secretion of profibrotic cytokines, myofibroblast generation, and inflammation [11,12]. Thus, it is important to determine factors that can protect against AKI and understand how to facilitate kidney recovery after injury, thereby preventing the transition to CKD.

The Hippo pathway is an evolutionarily conserved signaling cascade that regulates numerous biological processes [13,14]. Spanning across various organisms, this pathway serves as a fundamental mechanism for controlling critical cellular activities essential for maintaining tissue homeostasis and organ development [15]. The Hippo pathway plays a central role in coordinating diverse biological phenomena, from controlling cell proliferation and apoptosis to influencing tissue regeneration and organ size [13]. Its evolutionary conservation underscores its significance in governing cellular responses to environmental cues and developmental signals [16]. Through intricate molecular interactions and signaling events, the Hippo pathway ensures proper tissue architecture and function across different species, highlighting its importance in both normal physiological processes and pathological conditions [16]. Understanding the complexities of the Hippo pathway provides insights into the mechanisms that underlie health and disease, opening the door to potential therapeutic interventions aimed at this vital signaling network. Recent studies have implicated the Hippo pathway in the pathogenesis of AKI and its role in the repair process, as well as in the transition to CKD [14,17]. In in vivo studies, Hippo components play a protective role during AKI [14]. Salvador Family WW Domain Containing Protein 1 (SAV1) is a core component of the Hippo pathway, primarily involved in regulating organ size and tissue regeneration [15]. Recent studies have shown that loss of *SAV1* induces profibrotic gene expression and tubulointerstitial fibrosis in aristolochic acid-induced AKI and in unilateral ureteral obstruction [18,19]. However, the role of SAV1 in kidney IRI has yet to be defined. Here, we utilized proximal tubule-specific knockout of *SAV1* (*SAV1*^ptKO^) to demonstrate the role of SAV1 in kidney IRI.

## 2. Results

### 2.1. Distribution of SAV1 in Mouse Kidney Tuubles

**Figure 1:**
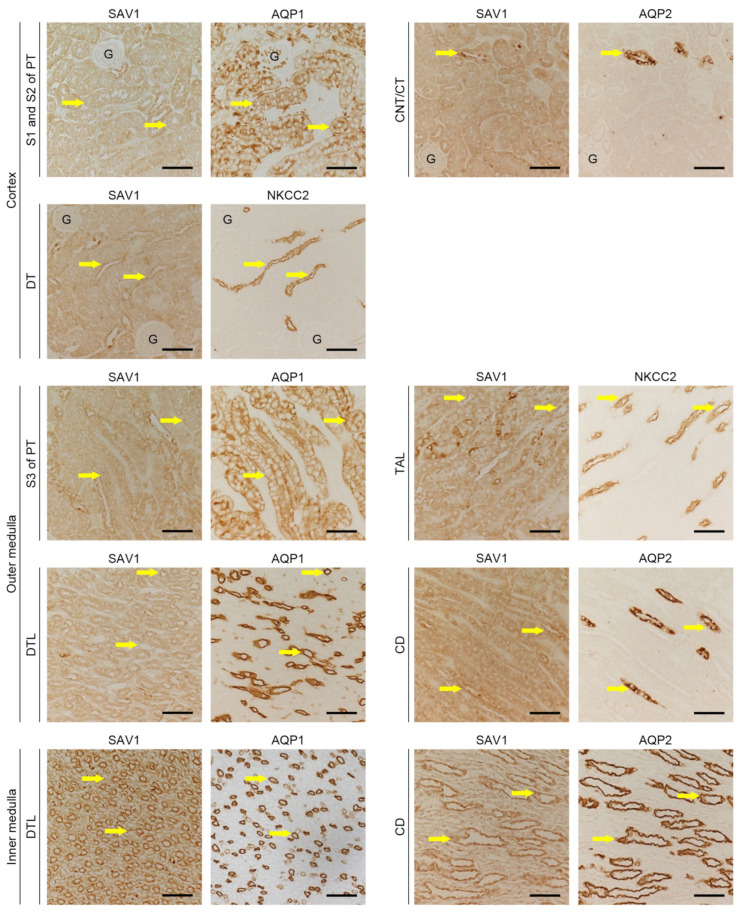
Expressions of SAV1 in kidney tubular cells. Sections of sham-operated kidneys were immunostained with antibodies against salvador family WW domain containing protein 1 (SAV1), aquaporin-1 (AQP1), aquaporin-2 (AQP2), and Na^+^-K^+^-Cl-cotransporter 2 (NKCC2). The antibody against AQP1 was used to label the S1, S2, and S3 segments of the proximal tubule (PT) and the descending thin limb of Henle’s loop (DTL). The antibody against AQP2 was used to label the connecting tubule (CNT), collecting tubule (CT), and collecting duct (CD). The antibody against NKCC2 was used to label the thick ascending limb of Henle’s loop (TAL) and the distal tubule (DT). Pictures were taken separately of the cortex, outer medulla, and inner medulla of the kidneys. Exemplary tubules are highlighted by arrows. G, glomerulus; scale bars, 50 μm.

The different segments of kidney tubules display unique responses under various physiological and pathophysiological conditions [20]. To determine the tubular localization of SAV1, immunohistochemistry was performed to stain for the protein within the kidney. Antibodies against aquaporin-1 (AQP1), aquaporin-2 (AQP2), and Na^+^-K^+^-Cl-cotransporter 2 (NKCC2) were used to identify tubular segments. As depicted in Figure 1, SAV1 expression was mainly observed in the cytoplasm of tubular cells in the inner medulla, with an intermediate level detected in the outer medulla, and the lowest level observed in the cortex. Upon closer examination, in the cortex, SAV1 expression was weak in the S1 and S2 segments of the proximal tubule (PT), intermediate in the distal tubule (DT), and strong in the connecting tubule (CNT) and the collecting tubule (CT). In the outer medulla, SAV1 expression was weak in the S3 segment of the PT and the descending thin limb of Henle’s loop (DTL), intermediate in the thick ascending limb (TAL), and strong in the collecting duct (CD). Ultimately, in the inner medulla, SAV1 was most robustly expressed in the CD, while its expression was faint in the DTL. These data indicate that SAV1 is expressed in all tubular cells with varying levels across different tubular segments.

### 2.2. Proximal Tubule-Specific SAV1 Deficiency Induces Maladaptive Restoration of Kidney Function after IRI

**Figure 2:**
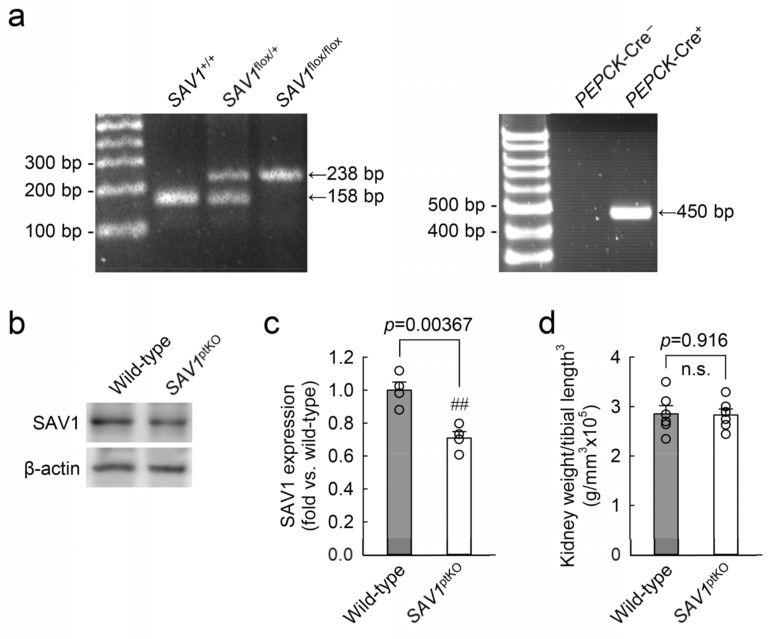
Generation of kidney proximal tubule-specific *SAV1* deficiency mice. Kidney proximal tubule-specific *SAV1* knockout mice (*SAV1*^ptKO^) were generated using *SAV1*^flox/flox^ mice and *PEPCK*-Cre transgenic mice (*PEPCK*-Cre^+^;*SAV1*^flox/flox^). (**a**) The left panel displays PCR genotyping of mice, showing the presence of the floxed allele (238 base pairs, bp) or the wild-type allele (158 bp). The right panel displays PCR genotyping of mice with *PEPCK*-Cre^+^ (450 bp) or without. (**b**) Representative Western blot analysis of SAV1 expression was presented. Anti-β-actin antibody was used as a loading control of the Western blot analysis. (**c**) The expression of SAV1 expression was quantified. (**d**) The ratio of whole kidney weight to tibial length^3^ was measured in wild-type and *SAV1*^ptKO^ mice. Statistical significance was determined by a 2-tailed unpaired Student’s *t*-test. ^##^ *p* < 0.01 vs. wild-type; n.s., no significant.

**Figure 3:**
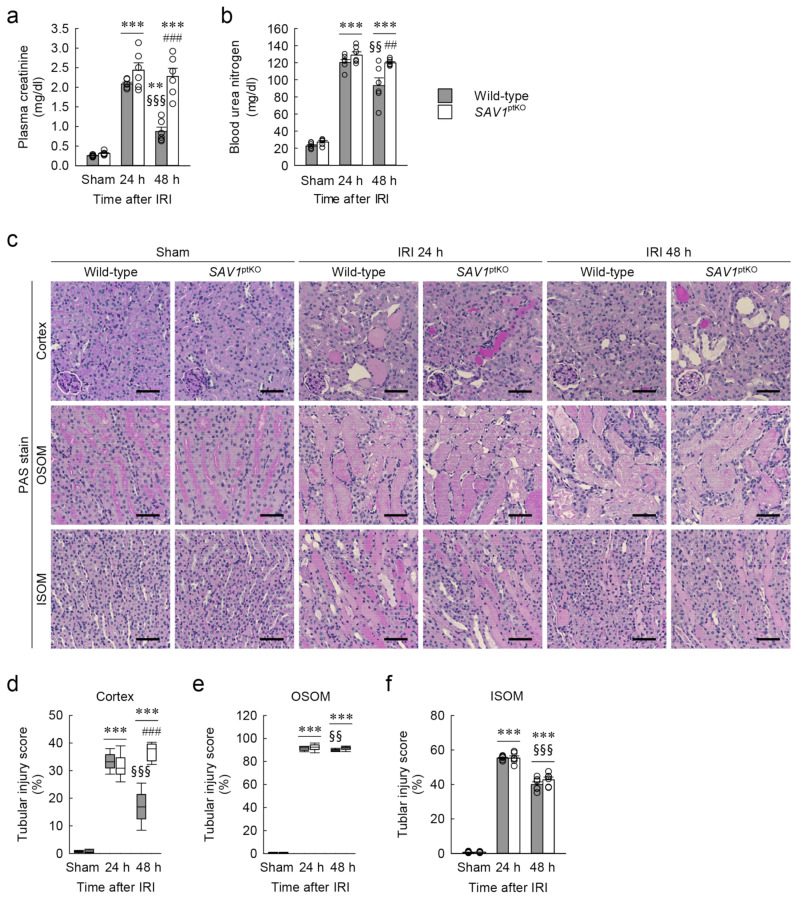
Loss of *SAV1* in kidney proximal tubules leads to maladaptive repair of both kidney function and structure after ischemia and reperfusion injury (IRI). Wild-type and *SAV1*^ptKO^ mice were subjected to 25 min of bilateral ischemia or sham operation, followed by 24 and 48 h of reperfusion. (**a**,**b**) Plasma creatinine and blood urea nitrogen concentrations were measured as indicators of kidney function. (**c**) Representative histology is shown by periodic acid-Schiff (PAS) stain in the cortex, outer stripe of the outer medulla (OSOM), and inner stripe of the outer medulla (ISOM) of the kidneys. Scale bar: 50 μm. (**d**–**f**) Tubular injury scores were assessed in the cortex, OSOM, and ISOM based on PAS-stained kidney sections. Statistical significance was determined by 2-way ANOVA followed by Tukey’s post hoc test (**a**,**b**) and the Kruskal–Wallis test followed by Student–Newman–Keuls post hoc test (**d**–**f**). ** *p* < 0.01, *** *p* < 0.001 versus sham; ^§§^ *p* < 0.01, ^§§§^ *p* < 0.001 vs. 24 h; ^##^ *p* < 0.01, ^###^ *p* < 0.001 vs. wild-type.

The genotype of SAV1^ptKO^ (SAV1^fl/fl^;PEPCK-Cre^+^) mice was confirmed through PCR-based genotyping of genomic DNA extracted from tail samples (Figure 2a). When the expression of SAV1 in both wild-type and SAV1^ptKO^ mice was examined using Western blot analysis, SAV1 expression was significantly decreased in SAV1^ptKO^ mice (Figure 2b,c). The data were analyzed using the 2-tailed unpaired Student’s *t*-test (t_6_ = 4.606, *p* = 0.00367). In another study, tamoxifen-induced deficiency of SAV1 in whole kidney tubules resulted in an increase in kidney size [18]. Due to this, the ratio of kidney weight to cubic tibial length was measured in both wild-type and SAV1^ptKO^ mice. There was no significant difference in the ratio between wild-type and SAV1^ptKO^ mice (Figure 2d), suggesting that SAV1 deficiency in kidney proximal tubules does not affect kidney size. The data were analyzed using the 2-tailed unpaired Student’s *t*-test (t_10_ = 0.108, *p* = 0.916). Next, to assess the functional impact of SAV1 deficiency in proximal tubules after IRI, kidney function in wild-type and SAV1^ptKO^ mice was evaluated by measuring both plasma creatinine and blood urea nitrogen (BUN) levels. As shown in Figure 3a,b, both parameters were significantly elevated at 24 and 48 h after IRI compared to levels in sham-operated mice. Particularly, at 48 h after IRI, the levels of plasma creatinine and BUN were significantly reduced compared to those measured at 24 h after IRI in wild-type mice, indicating the restoration of kidney function at 48 h (Figure 3a,b). However, the levels of plasma creatinine and BUN in SAV1^ptKO^ mice were not reduced at 48 h after IRI and were even significantly higher than those in wild-type mice (Figure 3a,b). Statistical analysis using 2-way analysis of variance (ANOVA) on plasma creatinine concentrations showed significant effects of IRI factor (*F*_2,30_ = 129.095, *p* < 0.001), SAV1^ptKO^ factor (*F*_1,35_ = 34.999, *p* < 0.001), and interaction between the two factors (*F*_2,35_ = 16.058, *p* < 0.001). Similarly, regarding BUN levels, the effects of IRI factor (*F*_2,30_ = 462.767, *p* < 0.001) and SAV1^ptKO^ factor (*F*_1,30_ = 12.550, *p* = 0.001) were significant, while interaction between both factors (*F*_2,35_ = 1.509, *p* = 0.237) was not significant. These findings suggest that proximal tubule-specific deficiency of SAV1 induces maladaptive restoration of kidney function after IRI.

### 2.3. Proximal Tubule-Specific SAV1 Deficiency Fails to Restore Cortical Tubular Structure after IRI

To assess the histological impact of *SAV1*^ptKO^ in IRI, the tubular injury score in both wild-type and *SAV1*^ptKO^ mice was evaluated on kidney sections stained with periodic acid-Schiff (PAS). As shown in Figure 3c–f, consistent with the kidney function results, tubular injury was notably elevated in the cortex, outer stripe of the outer medulla (OSOM), and inner stripe of the outer medulla (ISOM) at 24 and 48 h after IRI compared to the sham operation. Specifically, the tubular injury scores at 48 h after IRI showed a significant decrease in the cortex, the OSOM, and the ISOM compared to those observed at 24 h after IRI, suggesting the initiation of tubular structure restoration at 48 h in wild-type mice (Figure 3c–f). However, *SAV1*^ptKO^ mice at 48 h showed that the increased tubular injury score in the cortex was not reduced and was even significantly higher than that in wild-type mice (Figure 3d). The tubular injury score in the OSOM in the kidneys of *SAV1*^ptKO^ mice at 48 h was also not significantly reduced compared to that at 24 h (Figure 3e). Statistical analysis using the Kruskal–Wallis test on tubular injury scores in the cortex and the OSOM showed significant differences among the groups: (d) *H* = 30.641, *N*_1–6_ = 6, *p* ≤ 0.001; (e) *H* = 25.527, *N*_1–6_ = 6, *p* ≤ 0.001). Two-way ANOVA of tubular injury score in the ISOM revealed a significant effect of IRI factor (*F*_2,30_ = 1494.635, *p* < 0.001), while the effects of *SAV1*^ptKO^ factor (*F*_1,30_ = 1.230, *p* = 0.276) and interaction between both factors (*F*_2,30_ = 1.122, *p* = 0.339) were not significant. These findings suggest that a proximal tubule-specific deficiency of *SAV1* induces maladaptive restoration of the cortical tubular structure after IRI.

### 2.4. SAV1 Deficiency in Proximal Tubules Delays Cell Proliferation in the Cortex after IRI

**Figure 4:**
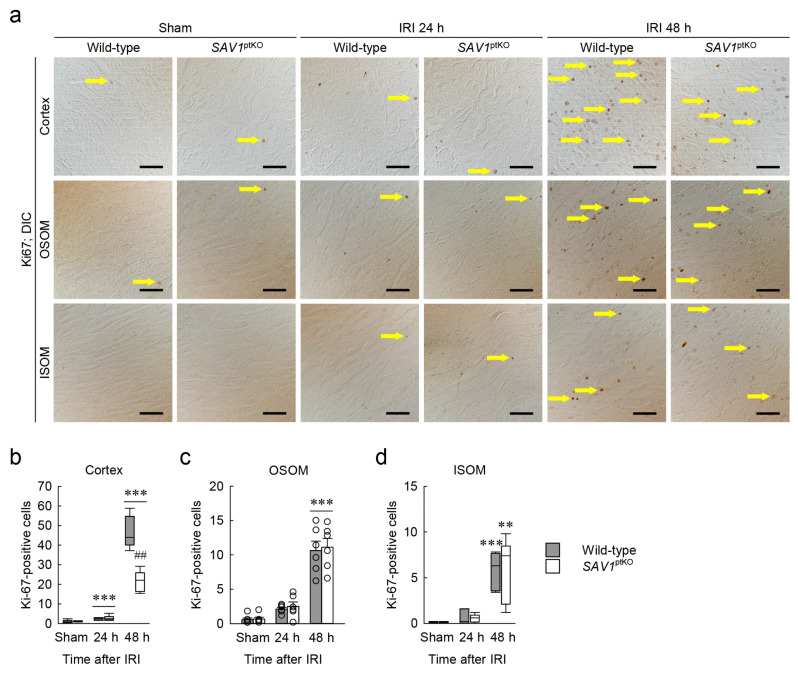
Loss of S*AV1* in kidney proximal tubules decreases cell proliferation after IRI. Wild-type and *SAV1*^ptKO^ mice were subjected to 25 min of bilateral ischemia or sham operation, followed by 24 and 48 h of reperfusion. (**a**) Representative immunohistochemistry of Ki67 was conducted in the cortex, outer stripe of the outer medulla (OSOM), and inner stripe of the outer medulla (ISOM) of the kidneys. Arrows indicate Ki67-positive cells. Scale bars: 50 μm. (**b**–**d**) The numbers of Ki67-positive cells in the cortex, OSOM, and ISOM were counted in high-power (400×) fields. Statistical significance was determined by the Kruskal–Wallis test followed by the Student–Newman–Keuls post hoc test (**b**,**d**) and 2-way ANOVA followed by Tukey’s post hoc test (**c**). ** *p* < 0.01, *** *p* < 0.001 versus sham; ^##^ *p* < 0.01 vs. wild-type.

To investigate the role of SAV1 in the proximal tubule regarding cell proliferation in kidneys subjected to IRI, the kidney of wild-type and *SAV1*^ptKO^ mice were stained with Ki67, a marker for cell proliferation, and the number of positive cells was quantified. As shown in Figure 4a–d, immunohistochemistry revealed a higher concentration of Ki67-positive cells in the cortex of kidneys at 48 h after IRI compared to the OSOM and the ISOM. This indicates that the cortex is the first to undergo restoration after tubular injury induced by IRI. However, at 48 h after IRI, the number of Ki67-positive cells in the cortex of *SAV1*^ptKO^ mice was significantly lower than that in wild-type mice (Figure 4b). The numbers in other regions of the kidney were not significantly different between wild-type and *SAV1*^ptKO^ mice (Figure 4c,d). Statistical analysis using the Kruskal–Wallis test of the number of Ki67-positive cells in the cortex and the ISOM showed significant differences among the groups: (b) *H* = 31.232, *N*_1–6_ = 6, *p* ≤ 0.001; (d) *H* = 25.593, *N*_1–6_ = 6, *p* ≤ 0.001). Two-way ANOVA of the number of Ki67-positive cells in the OSOM revealed a significant effect of IRI factor (*F*_2,30_ = 87.344, *p* < 0.001), while the effect of *SAV1*^ptKO^ factor (*F*_1,35_ = 0.120, *p* = 0.650) and interaction between both factors (*F*_2,30_ = 0.0260, *p* = 0.974) were not significant. These findings suggest that a proximal tubule-specific deficiency of *SAV1* delays cell proliferation in the cortex after IRI.

### 2.5. SAV1 in Proximal Tubules Plays a Role beyond the Hippo Pathway in Kidneys Exposed to IRI

**Figure 5:**
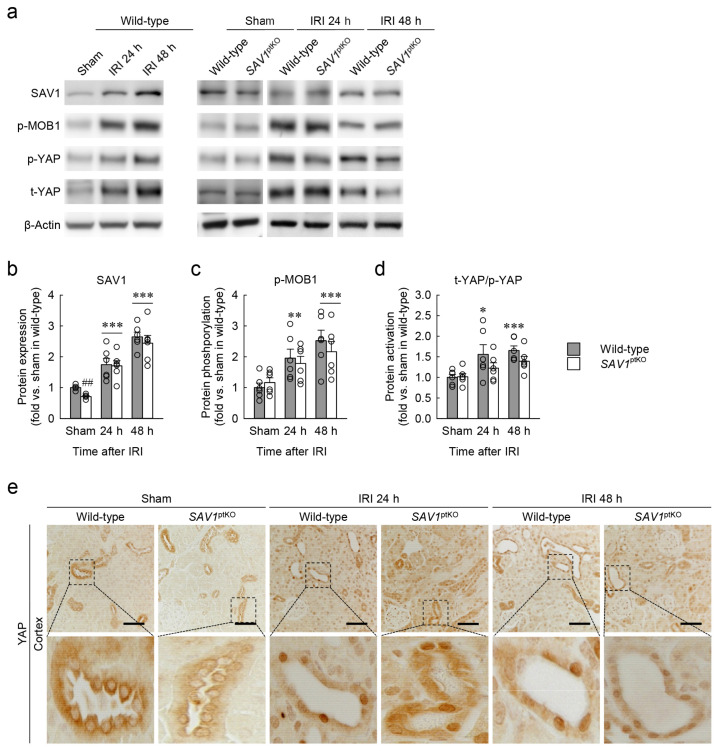
Maladaptive repair induced by loss of *SAV1* in kidney proximal tubules is independent of the Hippo pathway following kidney IRI. Wild-type and *SAV1*^ptKO^ mice were subjected to 25 min of bilateral ischemia or sham operation, followed by 24 and 48 h of reperfusion. (**a**) Representative Western blot analysis of SAV1, phosphorylated Mps-one binder kinase activator 1 (p-MOB1), phosphorylated yes-associated protein (p-YAP), and total YAP (t-YAP) expression were presented. (**b**–**d**) Quantification of SAV1 expression, MOB1 phosphorylation, and YAP activation. (**e**) Representative immunohistochemistry of YAP in the kidney cortex was presented. Scale bar: 50 μm. Statistical significance was determined by 2-way ANOVA followed by Tukey’s post hoc test. * *p* < 0.05, ** *p* < 0.01, *** *p* < 0.001 versus sham; ^##^ *p* < 0.01 vs. wild-type.

SAV1 is a WW domain containing protein originally identified as one of the core components of the Hippo pathway [21]. To determine whether SAV1 in proximal tubules functions on the Hippo pathway in kidneys subjected to IRI, the expressions of Hippo pathway-related proteins were assessed using Western blot analysis. SAV1 expression was increased over time after IRI compared to that in sham-operated kidneys, but there was no significant difference between wild-type and *SAV1*^ptKO^ mice (Figure 5a,b). Statistical analysis using 2-way ANOVA on SAV1 expression revealed a significant effect of IRI factor (*F*_2,26_ = 34.97, *p* < 0.001), while *SAV1*^ptKO^ factor (*F*_1,26_ = 0.754, *p* = 0.393) and interaction between the two factors (*F*_2,26_ = 0.109, *p* = 0.897) were not significant. Next, phosphorylation of Mps-one binder kinase activator 1 (MOB1) was significantly increased at 24 and 48 h, with no significant differences observed between wild-type and *SAV1*^ptKO^ mice (Figure 5a,c). On MOB1 phosphorylation, the effect of IRI factor was significant (*F*_2,30_ = 11.917, *p* < 0.001), while *SAV1*^ptKO^ factor (*F*_1,30_ = 0.313, *p* = 0.580) and interaction between the two factors (*F*_2,30_ = 0.518, *p* = 0.601) were not significant. Finally, activation of the yes-associated protein (YAP), as demonstrated by the ratio of total YAP to phosphorylated YAP, was significantly increased at 24 and 48 h after IRI in wild-type mice, but not in *SAV1*^ptKO^ mice (Figure 5a,d). Two-way ANOVA on YAP activation showed a significant effect of IRI factor (*F*_2,30_ = 7.958, *p* = 0.002), while *SAV1*^ptKO^ factor (*F*_1,30_ = 3.063, *p* = 0.090) and interaction between the two factors (*F*_2,30_ = 0.928, *p* = 0.406) were not significant. In addition to protein expression analysis conducted through Western blot analysis, immunohistochemisty was utilized to determine the cellular localization of YAP. The active form of YAP, which is localized in the nucleus, is the dephosphorylated form, while the phosphorylated form is localized in the cytoplasm [22]. YAP was detected in the cytoplasm of tubules in sham-operated kidneys, but it translocated into the nucleus of tubules at 24 and 48 h after IRI (Figure 5e). These findings suggest that SAV1 in proximal tubules plays a role beyond the Hippo signaling pathway in kidneys exposed to IRI.

### 2.6. Loss of SAV1 in the Kidney Proximal Tubule Activates AKT after Kidney IRI

**Figure 6:**
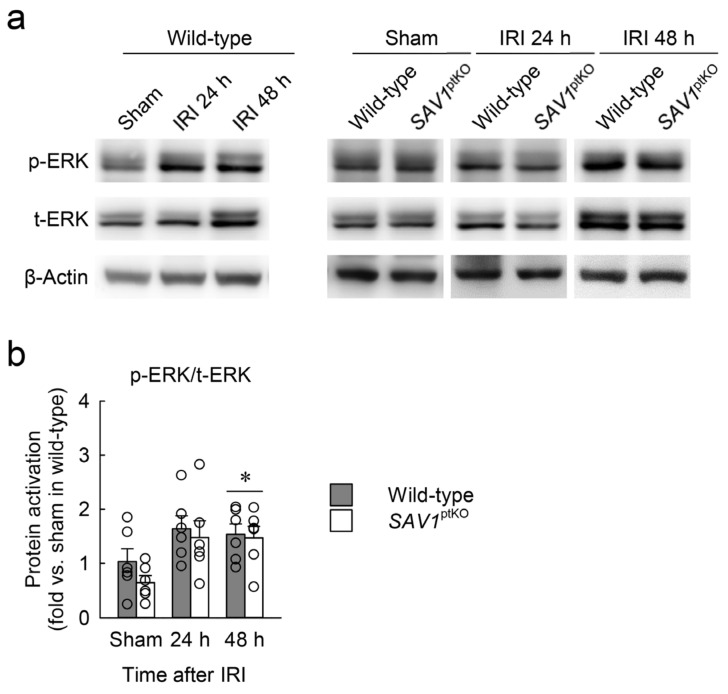
Loss of *SAV1* in the kidney proximal tubules is not associated with extracellular signal-regulated kinase (ERK) activation after IRI. Wild-type and *SAV1*^ptKO^ mice were subjected to 25 min of bilateral ischemia or sham operation, followed by 24 and 48 h of reperfusion. (**a**) A representative Western blot analysis of phosphorylated ERK (p-ERK) and total ERK (t-ERK) expression was presented. (**b**) The quantification of ERK activation was presented. Statistical significance was determined by 2-way ANOVA followed by Tukey’s post hoc test. * *p* < 0.05 vs. sham.

It is well-known that kidney IRI increases mitogen-activated protein kinase (MAPK) activation, and extracellular signal-regulated kinase (ERK), among MAPKs, contributes to cell proliferation after various tissue damage [23]. To investigate the association between SAV1 in proximal tubules and ERK activation following IRI, ERK phosphorylation and expression were assessed using Western blot analysis. As shown in Figure 6a,b, both the phosphorylation and expression of ERK were elevated in kidneys subjected to IRI. Furthermore, ERK activation significantly increased at 48 h after IRI, but there was no significant difference between wild-type and *SAV1*^ptKO^ mice (Figure 6a,b). Statistically, the effect of IRI factor on ERK activation was significant (*F*_2,30_ = 6.192, *p* = 0.006), while *SAV1*^ptKO^ factor (*F*_1,30_ = 1.196, *p* = 0.283) and interaction between the two factors (*F*_2,30_ = 0.268, *p* = 0.767) were not significant. These findings suggest that delayed cell proliferation after IRI in the kidneys of *SAV1*^ptKO^ mice is not linked to ERK activation.

**Figure 7:**
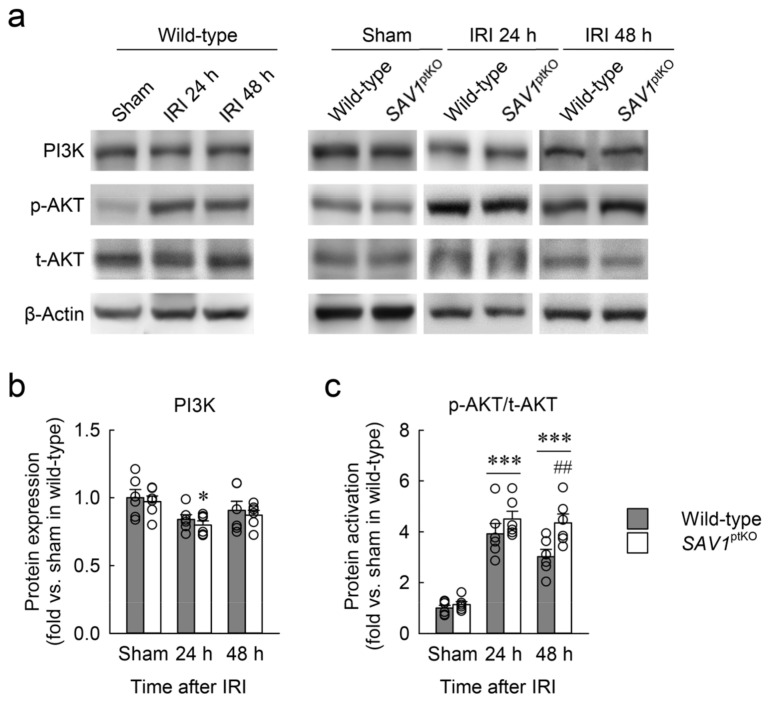
*SAV1* deletion in renal proximal tubules prolongs post-ischemic protein kinase B (AKT) activation. Wild-type and *SAV1*^ptKO^ mice were subjected to 25 min of bilateral ischemia or sham operation followed by 24 and 48 h of reperfusion. (**a**) A representative Western blot analysis displaying the expression levels of phosphoinositide 3-kinase (PI3K), phosphorylated AKT (p-AKT), and total AKT (t-AKT) was presented. (**b**,**c**) The expression of PI3K expression and activation of AKT were quantified. Statistical significance was determined by 2-way ANOVA followed by Tukey’s post hoc test. * *p* < 0.05, *** *p* < 0.001 vs. sham; ^##^ *p* < 0.01 vs. wild-type.

AKT plays an essential role in regulating cell proliferation [24]. To investigate the association between SAV1 in proximal tubules and AKT activation following IRI, phosphoinositide 3-kinase (PI3K) expression and protein kinase B (AKT) activation were assessed using Western blot analysis. PI3K expression was slightly decreased at 24 h after IRI compared with the sham operation, and its expression showed no significant alterations in both wild-type and *SAV1*^ptKO^ mice (Figure 7a,b). However, AKT activation was significantly increased at 24 and 48 h after IRI compared with the sham operation (Figure 7a,c). Furthermore, at 48 h after IRI, AKT activation in the kidneys of *SAV1*^ptKO^ mice was significantly higher than that in the wild-type mice (Figure 7a,c). Statistical analysis of PI3K expression revealed a significant effect of IRI factor (*F*_2,30_ = 6.268, *p* = 0.005), while *SAV1*^ptKO^ (*F*_1,30_ = 0.816, *p* = 0.373) and interaction between the two factors (*F*_2,30_ = 0.00669, *p* = 0.993) were not significant. On AKT activation, the effects of IRI factor (*F*_2,30_ = 70.796, *p* < 0.001) and *SAV1*^ptKO^ factor (*F*_1,30_ = 8.647, *p* = 0.006) were significant, while interaction between the two factors (*F*_2,30_ = 2.238, *p* = 0.124) was not significant. These findings suggest that a proximal tubule-specific deficiency of SAV1 contributes to AKT activation during the restoration period after IRI.

## 3. Discussion

**Figure 8:**
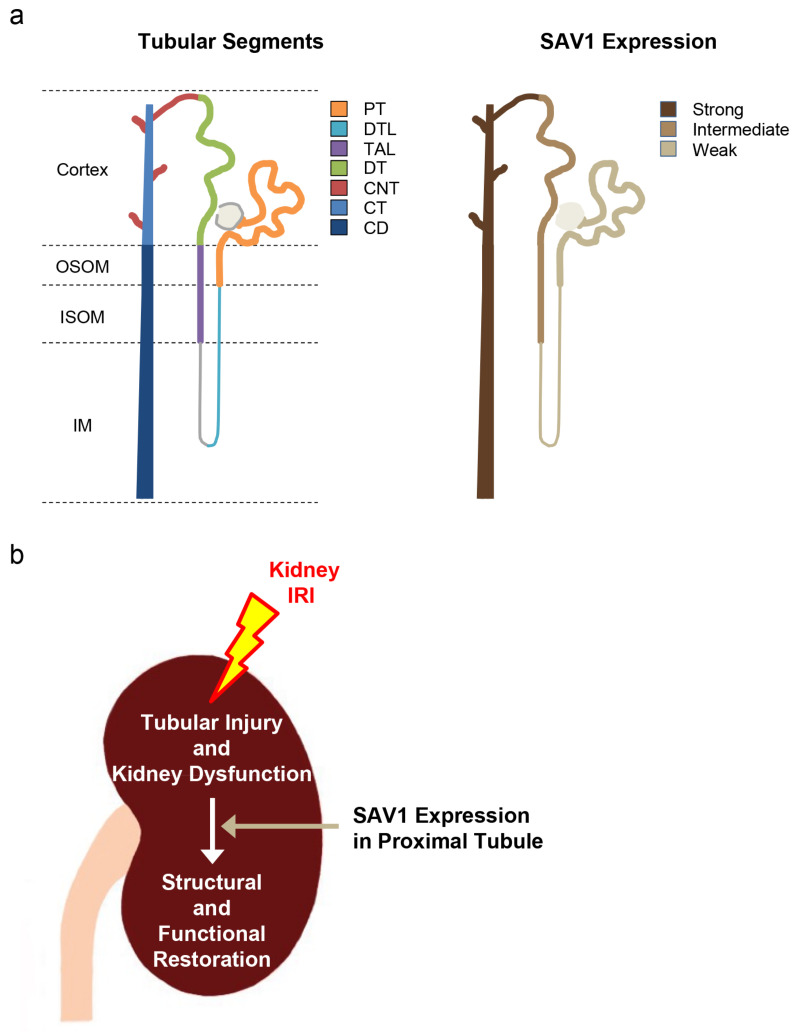
Schemes of SAV1 expression along tubular segments (**a**) and the mechanism of kidney proximal tubular SAV1 in kidneys subjected to IRI (**b**).

Kidney IRI is a common cause of AKI, impacting individuals of various species, genders, and ages [25,26,27]. Despite advancements in treatment, the mortality rate associated with AKI remains high. AKI poses a risk for the development and progression of CKD, potentially leading to end-stage renal disease. Recently, research has uncovered that maladaptive repair is a complex interplay involving various factors such as cell death, endothelial dysfunction, tubular epithelial cell dysfunction, inflammatory processes, and more, ultimately culminating in fibrosis [28]. Thus, it is crucial to explore strategies for preventing AKI and promoting kidney restoration after IRI. Indeed, the mechanisms underlying how SAV1 contributes to the restoration of kidney injury after IRI are not well understood and warrant further investigation. In the current study, it is demonstrated, for the first time, to our knowledge, the following key findings: (1) SAV1 exhibits its lowest expression in the PT and highest expression in the CNT to the CD (Figure 8a); (2) *SAV1* deficiency in kidney proximal tubules does not affect kidney size; (3) *SAV1* in kidney proximal tubules restores tubular structure and function following kidney IRI; (4) proximal tubule-specific *SAV1* deficiency results in a delay in cell proliferation in the renal cortex of kidneys subjected to IRI (Figure 8b); (5) proximal tubule-specific *SAV1* deficiency causes overactivation of AKT following kidney IRI.

Despite significant progress in elucidating the biological functions of the Hippo pathway, the specific role of SAV1 in kidney IRI has remained elusive. In our study, we discovered that SAV1 was predominantly expressed in kidney tubules, with a particular emphasis on the CNT to the CD. Kidney IRI is associated with the generation of reactive oxygen species, cytokines, and chemokines, all of which are abundantly produced during IRI due to a reduction in blood flow [29]. Consequently, various tubules in the OSOM are damaged. Notably, the proximal tubules, especially the S3 segment located in the OSOM, are particularly susceptible to severe damage during IRI [30]. Due to this, we generated mice with kidney proximal tubular cell-specific *SAV1* knockout using the *PEPCK*-Cre mouse line, representing *SAV1*^ptKO^ mice. In vivo studies revealed an increase in organ size upon the specific excision of *SAV1* [18,31]. Our data, however, show that the kidney weight-to-tibial length^3^ ratio did not exhibit a significant change between wild-type and *SAV1*^ptKO^ mice. Consistent with the current findings, the kidney weight of *SAV1* knockout mice in whole kidney tubules did not differ significantly from wild-type mice [32]. Thus, there is no observable change in gross phenotype between wild-type and *SAV1*^ptKO^ mice.

IRI is a common cause of kidney dysfunction, and the deterioration of kidney function is associated with increased morbidity and mortality in various diseases [33]. Creatinine, a representative indicator of kidney function, is a waste product produced in muscles and is mostly excreted through the kidneys [34]. Another indicator of kidney function is BUN [35]. In the current study, levels of creatinine and BUN were increased in mice that underwent kidney IRI, indicating induced kidney dysfunction. However, 48 h after IRI, creatinine and BUN levels were significantly lower in wild-type mice than *SAV1*^ptKO^ mice. The mechanisms underlying kidney restoration after IRI involve the loss of the brush border, mainly in the proximal tubular epithelium, and epithelial cell sloughing, followed by the repopulation of the damaged tubules by regenerating cells [36]. Consistent with the kidney function data, the current study showed that 48 h after IRI, kidney tubular damage in the cortex significantly decreased in wild-type mice compared to *SAV1*^ptKO^ mice. Complete kidney restoration involves addressing both structural and functional damage. Mouse studies have shown that tubular restoration after AKI is primarily, if not solely, facilitated by the regeneration of surviving proximal tubule cells [37]. Hence, our data demonstrate delayed kidney restoration following kidney IRI in *SAV1*^ptKO^ mice.

Critical factors influencing the integrity and recovery of injured nephrons include tubular epithelial cell proliferation and death [38]. Examining the expression of Ki67, a cell proliferation marker, can provide insights into the potential for recovery from tubular brush border loss, flattening, and nuclear pyknosis [39]. The current results indicate that the cortex in wild-type kidneys at 48 h after IRI had a greater concentration of Ki67-positive cells than *SAV1*^ptKO^ mice, suggesting that the cortex is the primary site of kidney damage restoration induced by IRI. Consistent with our results, in an in vivo model using B cell-deficient mice, an increase in Ki67-postive cells was observed after kidney IRI, leading to reduced tubular injury [40]. Kidney tubular cells in the cortex of IRI kidneys, after blood flow has returned to near-normal levels, have been suggested as an early site of repair processes during AKI [40,41].

The Hippo pathway, an evolutionarily conserved protein kinase cascade, plays a critical role in controlling organ size, cancer development, and tissue regeneration. The core components of this pathway in mammalian cells include mammalian Ste20-like kinase 1/2 (MST1/2) and SAV1. Their complex activates large tumor suppressor 1/2 (LAST1/2), MOB1, and p-YAP or phosphorylated transcriptional coactivator with PDZ-binding motif (TAZ). MST1/2 and SAV1 can phosphorylate LATS1/2 and MOB1. The activation of LATS1/2 and MOB1 can then phosphorylate YAP [13,16]. The Hippo pathway has been implicated in mediating kidney dysfunction after AKI [14,17]. In the current study, it has been observed that IRI significantly induced Hippo pathway-related proteins in whole kidneys and increased the number of YAP-positive cells in the nucleus. However, there were no significant differences between WT and *SAV1*^ptKO^ mice. Consistent with the current results, YAP activity was not altered following loss of *SAV1* in mouse livers [42]. Thus, proximal tubular SAV1 is not associated with the Hippo signaling pathway following kidney IRI.

Tubular damage induced by kidney IRI significantly enhanced ERK and AKT activation [43,44]. The ERK pathway mediates various cellular fates, including growth, proliferation, and survival. Here, it has been demonstrated that IRI markedly increases ERK phosphorylation, expression, and activation in whole kidneys. However, no significant differences were observed between wild-type and *SAV1*^ptKO^ mice. On the other hand, the AKT family consists of serine/threonine protein kinases that play critical roles in regulating growth, proliferation, survival, metabolism, and other cellular activities. However, chronic cellular stress and prolonged phosphorylation, leading to supraphysiological activation of AKT, may increase oxidative stress and contribute to oxidative injury [45]. AKT activation is also a crucial node in various signaling cascades involved in kidney damage [46]. In the current study, we observed that AKT activation increased as a result of proximal tubule-specific *SAV1* deficiency at 48 h after kidney IRI. These data, along with previous studies, suggest that loss of *SAV1* in the proximal tubules induces AKT activation during IRI, exacerbating the impairment of kidney restoration. Consistent with the current results, chronic AKT activation has been shown to adversely affect the heart’s response to ischemia [47]. Another study has reported a significant increase in kidney AKT activation in a model of tubulointerstitial fibrosis induced by unilateral ureteral obstruction [46]. Thus, *SAV1* deficiency in the proximal tubules delays kidney restoration by excessively activating AKT.

## 4. Conclusions

In conclusion, the current study has shown that the *SAV1* deficiency in the kidney proximal tubule induces maladaptive repair after kidney IRI through AKT activation. Nevertheless, additional studies are needed to ascertain the direct association between AKT activation resulting from *SAV1* deficiency in the kidney proximal tubule and maladaptive impairment. This finding provides a novel therapeutic strategy for preventing maladaptive repair induced by kidney IRI.

## 5. Materials and Methods

### 5.1. Animal Preparation

Homozygous *SAV1*-floxed mice (*SAV1*^fl/fl^) were provided by Dr. Dae-Sik Lim from the Korea Advanced Institute of Science and Technology in Daejeon, Republic of Korea [48]. The breeding strategy for transgenic mice expressing Cre recombinase under the control of the kidney-specific *PEPCK* promoter (*PEPCK*-Cre) was reported elsewhere [49]. To generate *SAV1*^ptKO^ mice, *PEPCK*-Cre-positive mice (*PEPCK*-Cre^+^) and *SAV1*^fl/fl^ were utilized. A routine polymerase chain reaction genotyping from tail DNA samples used the following primer pairs: *SAV1*, 5′-TGCTGGTTTTGTCTCACTAA-3′ and 5′-TGGTTTGCTTTTTAGTGGCC-3′; *Cre*, 5′-CGGTGCTAACCAGCGTTTTC-3′ and 5′-TGGGCGGCATGGTGCAAGTT-3′. Male littermates of homozygous *SAV1*-floxed and *PEPCK*-Cre-negative genotype (*SAV1*^fl/fl^;*PEPCK*-Cre^−^) were utilized as wild-type controls. All mice were born at the expected Mendelian frequency and did not display any gross physical or behavioral abnormalities. The mice were also housed under a 12 h light/dark cycle at 22 ± 2 °C with 55 ± 5% humidity and had unrestricted access to water and standard mouse diet chow.

### 5.2. Surgery

All mouse experiments were conducted in compliance with animal protocols approved by the Institutional Animal Care and Use Committee of Jeju National University (approval no. 2023-0038 and 2021-0045). Kidney IRI was induced following previously described methods [50]. Briefly, 8- to 10-week-old male mice were anesthetized by intraperitoneal injection of pentobarbital sodium (ENTOBAR; 60 mg/kg body weight; Hanlim Pharm, Seoul, Republic of Korea). During kidney ischemia, the mouse’s body temperature was maintained at 36.5 °C to 37.5 °C using a DC temperature control system (FHC, Bowdoin, ME, USA). After exposing the kidneys through flank incisions, the mice underwent 25 min of bilateral ischemia by clamping the renal pedicles using micro clips (Roboz, Rockville, MD, USA; catalog no. RS-5424) and micro clip applying forceps (Roboz; catalog no. RS-5440), followed by 24 and 48 h of reperfusion. During ischemic surgery, the incisions were temporarily closed using Hartman hemostats (Fine Science Tools, Foster City, CA, USA; catalog no. 13002-10). After the clamps were removed, reperfusion of the kidneys was visually confirmed. After confirming reperfusion, the skin was closed using autoclip applier (Fine Science Tools; catalog no. 12020-09 100), and the mice were allowed to recover on a warming pad until they were fully awake. The respective mortalities 24 and 48 h after IRI were 100% (n = 32) and 93.8% (n = 32), respectively. Sham-operated mice underwent the same surgical procedure without inducing ischemia. The left and right kidneys were randomly either snap-frozen in liquid nitrogen for Western blot analysis or fixed in 4% paraformaldehyde (Tech and Innovation, Chuncheon, Gangwon, Republic of Korea; catalog no. BPP-9016) for histological studies.

### 5.3. Kidney Function

At 24 and 48 h after IRI or sham operation, blood samples were collected from the retro-orbital sinus using heparinized capillary tubes (Kimble Chase, Vineland, NJ, USA; catalog no. 2501). The capillary tubes were centrifuged at 17,000× *g* for 10 min. The supernatants were used as plasma. The plasma concentrations of creatinine and BUN were measured using the respective QuantiChrom creatinine and urea assay kits (BioAssay Systems, Hayward, CA, USA; catalog no. DICT-500 and DICT-100), as previously described [50]. Briefly, the concentration of plasma creatinine was determined by transferring 30 µL of plasma into a 96-well plate. After adding 200 µL of working reagent (reagent A plus reagent B) to the 96-well plate, the absorbance was measured at 510 nm for 60 s using the SpectraMax i3x multi-mode microplate reader (Molecular Devices, San Jose, CA, USA) in the Bio-Health Materials Core-Facility at Jeju National University. The concentration of BUN was measured using the same process, with 5 µL of plasma, and the absorbance was read at 520 nm.

### 5.4. Immunohistochemistry

Immunohistochemical staining of the kidneys was performed on paraffin sections following previously described methods [50]. Briefly, kidney sections fixed in 4% paraformaldehyde were rehydrated and subjected to staining, including PAS, SAV1 (CUSABIO, Houston, TX, USA; catalog no. CSB-PA875672ESR2HU; 1:400 dilution), AQP1 (Alomone Labs, Jerusalem, Israel; catalog no. AQP-001, 1:500 dilution), AQP2 (Alomone Labs; catalog no. AQP-002, 1:1000 dilution), NKCC2 (Proteintech, Rosemont, USA; catalog no. 18970-1-AP, 1:2000 dilution), Ki67 antibody (Novus Biologicals, Littleton, CO, USA; catalog no. NB110-89717; 1:500 dilution), and YAP (Cell Signaling Technology, Danvers, MA, USA; catalog no. 4912; 1:200 dilution). For staining with the SAV1, AQP1, AQP2, NKCC2, Ki67, and YAP antibodies, the sections were incubated to peroxidase anti-rabbit IgG (Vector Laboratories, Burlingame, CA, USA; catalog no. PI-1000; 1:400 dilution) for 1 h at room temperature. The substrate reaction was induced using a DAB Substrate Kit (Vector Laboratories; catalog no. SK-4100) following the manufacturer’s instructions.

### 5.5. Tubular Injury and Cell Proliferation

The tubular injury score represents the percentage of injured tubules exhibiting cast formation, tubular dilation, and/or sloughing of cells on kidney sections stained with PAS, as previously described [50]. The score was evaluated in a blind manner using a Nikon Eclipse Ni microscope (Nikon, Tokyo, Japan). The numbers of total and injured tubules were counted in five randomly selected high-power fields (at 400× magnification) per kidney cortex, outer stripe of the outer medulla (OSOM), and inner stripe of the outer medulla (ISOM), respectively. To assess cell proliferation, the count of Ki67-positive cells was conducted blindly under the microscope [51]. Specifically, the count was performed in five randomly selected high-power fields (at 400× magnification) per kidney cortex, OSOM, and ISOM, respectively. After the counting, representative histomicrograph images were captured at high magnification using a DS-Ri2 camera (Nikon) and NIS-Elements imaging software (version 4.50, Nikon).

### 5.6. Western Blot Analysis

Kidney tissues were homogenized in ice-cold lysis buffer (Thermo Fisher Scientific, Waltham, MA, USA; catalog no. 78510). After centrifuging the homogenates at 17,000× *g* for 20 min at 4 °C, protein samples were extracted from the supernatants. The protein concentrations of the samples were assessed using Bradford reagent (Sigma-Aldrich, St. Louis, MO, USA; catalog no. B6916) and then mixed with a lane marker reducing sample buffer (Thermo Fisher Scientific; catalog no. 39000). The mixed protein samples were loaded onto 12% and 7.5% polyacrylamide gels prepared from TGX FastCast acrylamide kits (Bio-Rad Laboratories, Hercules, CA, USA; catalog no. 1610175 and 1610171, respectively) and subsequently transferred to polyvinylidene fluoride membranes (Bio-Rad Laboratories), following previously established protocols [50,52]. The membranes were incubated with antibodies against SAV1 (Cell Signaling Technology; catalog no. 13301; 1:5000 dilution), phosphorylated MOB1 (p-MOB1; Cell Signaling Technology; catalog no. 8699S; 1:5000 dilution), phosphorylated YAP (p-YAP; Cell Signaling Technology; catalog no. 13008; 1:5000 dilution), total YAP (t-YAP; Cell Signaling Technology; catalog no. 4912; 1:5000 dilution), PI3K (Cell Signaling Technology; catalog no. 4292; 1:2500 dilution), phosphorylated AKT (p-AKT; ABclonal Technology, Woburn, MA, USA; catalog no. AP0637; 1:2500 dilution), total AKT (t-AKT; Santa Cruz Biotechnology, Santa Cruz, CA, USA; catalog no. sc-8312; 1:2500 dilution), phosphorylated ERK (p-ERK; Cell Signaling Technology; catalog no. 4370; 1:5000 dilution), total ERK (t-ERK; Santa Cruz Biotechnology; catalog no. sc-93; 1:10,000 dilution), and β-actin (Santa Cruz Biotechnology; catalog no. sc-47778; 1:5000 dilution) overnight at 4 °C. After washing, peroxidase anti-rabbit IgG antibody (Vector Laboratories; catalog no. WB-1000; 1:5000 dilution) against SAV1, p-MOB1, p-YAP, t-YAP, PI3K, p-AKT, t-AKT, p-ERK, and t-ERK antibodies, and peroxidase anti-mouse IgG antibody (1:5000 dilution; catalog no. WB-2000; Vector Laboratories) against β-actin antibodies were applied for 60 min at room temperature. Subsequently, Western Lightning chemiluminescence reagent (PerkinElmer, Boston, MA, USA; catalog no. NEL101) was used to detect proteins in the Azure c300 imaging system (Azure Biosystems, Dublin, CA, USA). The quantification of the detected protein bands was performed using AzureSpot analysis software version 14.2 (Azure Biosystems).

### 5.7. Kidney Size

After the sacrifice of the mice, the kidney weight was immediately measured using an analytical balance (Sartorius, Goettingen, Germany; catalog no. BP210S). After the dissection of both tibiae, the tibial length was measured vertically from the medial tibial condyle to the medial malleolus using a vernier caliper (BLUETEC, Goyang, Gyeonggi, Republic of Korea). Subsequently, the kidney-to-tibial length^3^ ratio was calculated to identify the kidney size, as previously described [53].

### 5.8. Statistical Analysis

All statistical analyses of the data were conducted using SigmaPlot 14.0 software (Systat Software Inc., San Jose, CA, USA), as previously described [50]. The normal distribution was assessed using the Shapiro–Wilk normality test. In cases where the data did not exhibit a normal distribution, an attempt was made to apply logarithmic transformation. Parametric data passing the normality test were analyzed with 2-way ANOVA followed by Tukey’s post hoc test. In ANOVA, the notation F_α,β_ = γ represents the degrees of freedom for explained variance (α), for residual variance (β), and the *F* value (γ). Non-parametric data that did not pass the normality test were analyzed using the Kruskal–Wallis test followed by the Student–Newman–Keuls post hoc test. In the Kruskal–Wallis test, the notation *H* = α, *N*_β_ = γ represents the *H* value (α), the number of groups (β), and the sample size (γ). Statistical significance between two groups was determined by 2-tailed unpaired Student’s *t*-test with parametric data that passed an equal variance test. The equality of variances was assessed using the Brown–Forsythe test. In *t*_α_ = β for the *t* test, α and β represent the degrees of freedom and the *t*-value, respectively. In figures, parametric data are displayed as mean ± standard error of the mean (SEM) with individual data points, while non-parametric data are presented as median value with quartiles. A value of *p* < 0.05 was considered statistically significant. All raw numeric data and statistical results are provided in the Supplementary Materials.

## Data Availability

The data presented in this study are available from the corresponding author upon reasonable request.

## Supplementary Materials

The following supporting information on raw data and statistical results can be downloaded at: https://www.mdpi.com/article/10.3390/ijms25094610/s1.
